# Early stage periarticular injection during total knee arthroplasty may provide a better postoperative pain relief than late-stage periarticular injection: a randomized-controlled trial

**DOI:** 10.1007/s00167-018-5140-y

**Published:** 2018-09-20

**Authors:** Sachiyuki Tsukada, Kenji Kurosaka, Tetsuyuki Maeda, Akihiro Iida, Masahiro Nishino, Naoyuki Hirasawa

**Affiliations:** 1 Department of Orthopaedic Surgery, Hokusuikai Kinen Hospital, 3-2-1 Higashihara, Mito, Ibaraki 310-0035 Japan; 2 Department of Anesthesiology, Hokusuikai Kinen Hospital, Mito, Japan

**Keywords:** Primary knee arthroplasty, Pain, Pre-emptive analgesia, Periarticular injection, Injection technique, Randomized-controlled trial

## Abstract

**Purpose:**

This study was performed to determine whether periarticular injection performed in the early stage of total knee arthroplasty (TKA) could provide a better postoperative pain relief than periarticular injection performed in the late stage of TKA. The hypothesis was based on the concept that analgesic intervention before the onset of noxious stimuli would be associated with less postoperative pain.

**Methods:**

A total of 105 participants were randomly assigned to receive superficial injection just prior to arthrotomy (early stage periarticular injection group) or superficial injection after implanting the prosthesis (late-stage periarticular injection group) in patients undergoing unilateral TKA with 1:1 treatment allocation. In both groups, deep injection was performed according to the same schedule (just prior to implanting prosthesis). The solution consisted of 300 mg of ropivacaine, 8 mg of morphine, 40 mg of methylprednisolone, 50 mg of ketoprofen, and 0.3 mg of epinephrine mixed with normal saline to a final volume of 60 mL. All surgeries were managed under general anesthesia without any regional blocks. Registry-specified primary outcome was postoperative pain score at rest measured at the recovery room using a 100-mm visual analog scale (VAS). The VAS score was compared between two groups and assessed to reach the reported threshold values for the minimal clinically important difference (MCID) of 10 mm for the postoperative VAS score.

**Results:**

The VAS score at the recovery room was significantly lower in the early stage periarticular injection group than the late-stage periarticular injection group (23 ± 25 mm versus 39 ± 34 mm, respectively; 95% confidence interval 4–28 mm; *p* = 0.0078). The mean difference in the primary outcome fulfilled the MCID value.

**Conclusions:**

Bringing forward the timing of periarticular injection may provide significant and clinically meaningful improvement in pain following TKA under general anesthesia.

**Level of evidence:**

I.

## Introduction

Multimodal pain management has become standard practice to resolve severe pain after total knee arthroplasty (TKA) [12]. Periarticular injection is one of the most critical components of the multimodal pain management [12, 13, 21]. There has been a great deal of interest in effective techniques for periarticular injection.

Periarticular injection for TKA consists of superficial injection and deep injection [25]. Multi-drug solution is injected into the extensor mechanism, pes anserinus, and anteromedial capsule as superficial injection, and into the posterior capsule, posteromedial structures, and periarticular synovium as deep injection. In TKA managed with spinal anesthesia, both injections have been performed in the late stage of surgery; deep injection has been performed just before implantation and superficial injection just after implantation [2, 16, 24, 25].

In the setting of general anesthesia without any regional blocks, there has been a paucity of studies investigating the optimal timing of periarticular injection during TKA. Although the periarticular injection is becoming more common in orthopedic surgeries other than TKA [5, 9, 17], the optimal timing of periarticular injection also remains unclear in such surgeries. The concept of pre-emptive analgesia, in which analgesic intervention prior to the onset of noxious stimuli could reduce postoperative pain and opioid consumption, has been advocated [3, 8, 15]. To achieve pre-emptive analgesia, earlier analgesic intervention using periarticular injection may be associated with a better pain relief than the conventional technique of periarticular injection.

An investigator-initiated randomized-controlled trial was conducted to assess whether the periarticular injection performed in the early stage of surgery would reduce postoperative pain compared with the conventional periarticular injection performed in the late stage of surgery during TKA managed under general anesthesia without regional block. This is the first randomized-controlled trial to investigate the optimum timing of periarticular injection for postoperative pain relief after orthopedic surgery, and may contribute to improving postoperative pain relief only by changing the timing of the injection. The hypothesis of this study was that postoperative pain would be reduced by the early stage periarticular injection.

## Materials and methods

This prospective, two-arm, parallel-group, single-blinded randomized-controlled trial was performed in a single non-university hospital. The trial protocol was approved by the ethics committee of Hokusuikai Kinen Hospital (Registration Number R0010). Written informed consent to participate in the trial was obtained from each patient before randomization. The study was registered with the University Hospital Medical Information Network (Registration Number UMIN000021910) before enrollment of the first participant.

### Participants

Patients scheduled for TKA were invited to participate in this study by one treating surgeon (ST). Participants were recruited between September 2016 and December 2017. Eligible patients were at least 18 years of age and scheduled for unilateral TKA. Exclusion criteria were allergy or intolerance to one of the study drugs, or renal insufficiency.

Participants were informed that we were comparing a periarticular injection in the early stage of surgery with that in the late stage of surgery based on the concept of pre-emptive analgesia and they would be randomly assigned to receive periarticular injection in the early or late stage of surgery.

### Randomization and blinding

Using web-based randomization, participants were assigned in a 1:1 ratio to receive periarticular injection in the early stage of surgery or in the late stage of surgery. Randomized numbers from 0 to 99 were generated using a random number generator program available at http://www.random.org, by Randomness and Integrity Services Ltd., Ireland. Patients with even numbers were allocated to the early stage periarticular injection group, and those with odd numbers were allocated to the late-stage periarticular injection group.

Randomization results were transferred to the surgical team and anesthesiologist. The participants and nursing staff in the ward remained blinded to the allocation until 3 days after TKA. Staff members who had knowledge of the allocation were not involved in recording any primary outcome measures.

### Interventions

The study treatments were superficial injection in the early stage of TKA or in the late stage of TKA. All patients received deep injection just prior to implantation of the prosthesis.

In the early stage periarticular injection group, the superficial injection was performed just after exposing the fascial layer (just prior to arthrotomy).

In the late-stage periarticular injection group, the superficial injection was performed after implantation while waiting for the cement to cure.

The solution for periarticular injection consisted of 7.5-mg/mL ropivacaine (Anapeine, AstraZeneca, Osaka, Japan) (40 mL), 10-mg/mL morphine hydrochloride hydrate (Takeda, Osaka, Japan) (0.8 mL), 40-mg/mL methylprednisolone (Solu-Medrol, Pfizer, Tokyo, Japan) (1 mL), 20-mg/mL ketoprofen (Capisten, Kissei, Matsumoto, Japan) (2.5 mL), 1.0-mg/mL epinephrine (Bosmin, Daiichi-Sankyo, Tokyo, Japan) (0.3 mL), and normal saline (15.4 mL) [22, 23]. As superficial injection, 40 mL of solution was injected into the extensor mechanism, pes anserinus, retinaculum, and iliotibial band. As deep injection, 20 mL of the solution was injected into the posterior capsule and the posteromedial and posterolateral structures [22, 23].

### Pre- and postoperative medication

Antibiotic prophylaxis with 1 g of cefazolin (Cefamezin; Astellas, Tokyo, Japan) was intravenously administered perioperatively. To reduce perioperative blood loss, 1 g of tranexamic acid (Transamin; Daiichi-Sankyo) was administered intravenously just prior to skin incision.

No narcotic pain medications were used for postoperative medication. All patients received 4 mg of oral non-steroidal anti-inflammatory drug (lornoxicam; Lorcam, Taisho-Toyama, Tokyo, Japan) three times a day. A dose of 25 mg of non-steroidal anti-inflammatory drug suppository (diclofenac sodium suppository; Voltaren suppository; Novartis, Tokyo, Japan) was allowed as rescue analgesic medication.

### Anesthesia, surgery, and rehabilitation

All patients were managed with general anesthesia induced using short-acting volatile anesthetic (sevoflurane; Sevofrane, Maruishi, Tokyo, Japan) and intravenous anesthetic (propofol; Diprivan, AstraZeneca). The anesthesia was maintained with sevoflurane and continuous infusion of short-acting opioid (remifentanil, Ultiva; Janssen, Tokyo, Japan). The anesthesiologists performed supplementation with intravenous fentanyl citrate as needed (Fentanyl; Daiichi-Sankyo). The study protocol prohibited spinal anesthesia, regional anesthesia, and intraoperative administration of long-term narcotic analgesics except for the morphine included in the periarticular injection solution.

All surgical procedures were performed by one of two surgeons (ST and KK). No pneumatic tourniquet was used during study period. An anterolateral incision was used in all surgeries to reduce the amount of trauma to the branches of the saphenous nerve [20]. A subvastus approach was used except in patients with valgus knee alignment, for whom a lateral approach was used. All patients received a cemented, posterior-stabilized prosthesis (Persona; Zimmer-Biomet, Warsaw, IN). No drain was placed for any of the patients.

The postoperative rehabilitation regimen was the same for both groups. Physical therapy was begun the day after TKA. The patients were instructed to begin ambulating with assistance the day after TKA.

### Primary outcome

The prespecified primary outcome was the pain score at the recovery room. Pain score was rated using a 100-mm horizontal visual analog scale (VAS). The patients were told that the left end of a 100-mm line represented no pain and the right end represented the most extreme pain that they had ever felt. The patients were asked to put an X on the line in the place that best estimated their pain level at that moment. The VAS score was compared between groups as a continuous variable.

Despite its status as a gold standard to measure pain intensity, the VAS has the limitation that a reduction in a VAS score itself may not equate to improvement in the patient’s experience [6, 14]. Whether the difference in VAS score between groups reached the MCID and the value of VAS score fulfilled patient acceptable symptom state (PASS) were also assessed. The MCID is the smallest difference in an outcome score that a patient perceives as beneficial [6]. The PASS is the score below which patients consider themselves well [14, 19]. The VAS score at the recovery room was assessed to fulfill the prescribed VAS score published by Myles et al. [14]. Myles et al. concluded analgesic interventions that provided a change of 10 for the 100-mm pain VAS signified a clinically important improvement, and a VAS of 33 or less signified acceptable pain control after surgery [14].

Regarding test–retest reliability of the VAS pain score, Bijur et al. reported an intraclass correlation coefficient of 0.97 (95% confidence interval 0.96–0.98) in patients presenting to the emergency department with acute pain as a component of their chief complaint [1]. Myles et al. reported an intraclass correlation coefficient of 0.79 (95% confidence interval 0.49–0.91) in patients recovering from a surgical procedure requiring general or major neuraxial block anesthesia [14].

### Secondary outcomes

The pain scores at rest were also measured at 3 h, 6 h, 9 h, 12 h, 24 h, and 48 h after surgery.

Intraoperative blood loss was calculated as the sum of blood aspirated into the suction canisters and weighing gauzes.

The levels of remifentanil and fentanyl citrate consumption were recorded during surgery.

The number of suppositories used as postoperative rescue analgesia was recorded from the night of surgery to day 3 after TKA.

The range of knee movement was measured by the physiotherapist. The data were collected from days 1 to 7 after TKA.

Any complications were recorded during the trial.

### Statistical analyses

This randomized-controlled trial was planned as a superiority trial. Our primary hypothesis was that the early postoperative pain score among participants with superficial injection before arthrotomy would be better than that in patients who received superficial injection after implantation. The enrollment of 47 participants per treatment group was estimated to provide a power 80% to detect a between-group MCID of 10 mm in the VAS score [15, 18], at a type I error rate of 5%. For power analysis, we used a standard deviation of 17 mm in the VAS score for the data of a previous study in postoperative unilateral TKA patients [13].

The mean differences in the primary outcome and 95% confidence intervals were calculated with Student’s *t* test. The primary outcome was also compared using the Mann–Whitney *U* test. The complete-case analysis was performed for the missing data for the primary outcome [11]. Thus, participants with missing data were excluded from the analysis of primary outcome without imputation.

Comparisons between the study groups were performed with the Chi-squared test for categorical variables and Student’s *t* test for continuous variables. All tests were two-sided, and *p* < 0.05 was considered statistically significant.

All statistical analyses were performed with R software of version 3.3.2. (The R Foundation for Statistical Computing, Vienna, Austria) and EZR (Saitama Medical Center, Jichi Medical University, Saitama, Japan) [7].

## Results

### Enrollment and patient characteristics

**Fig. 1:**
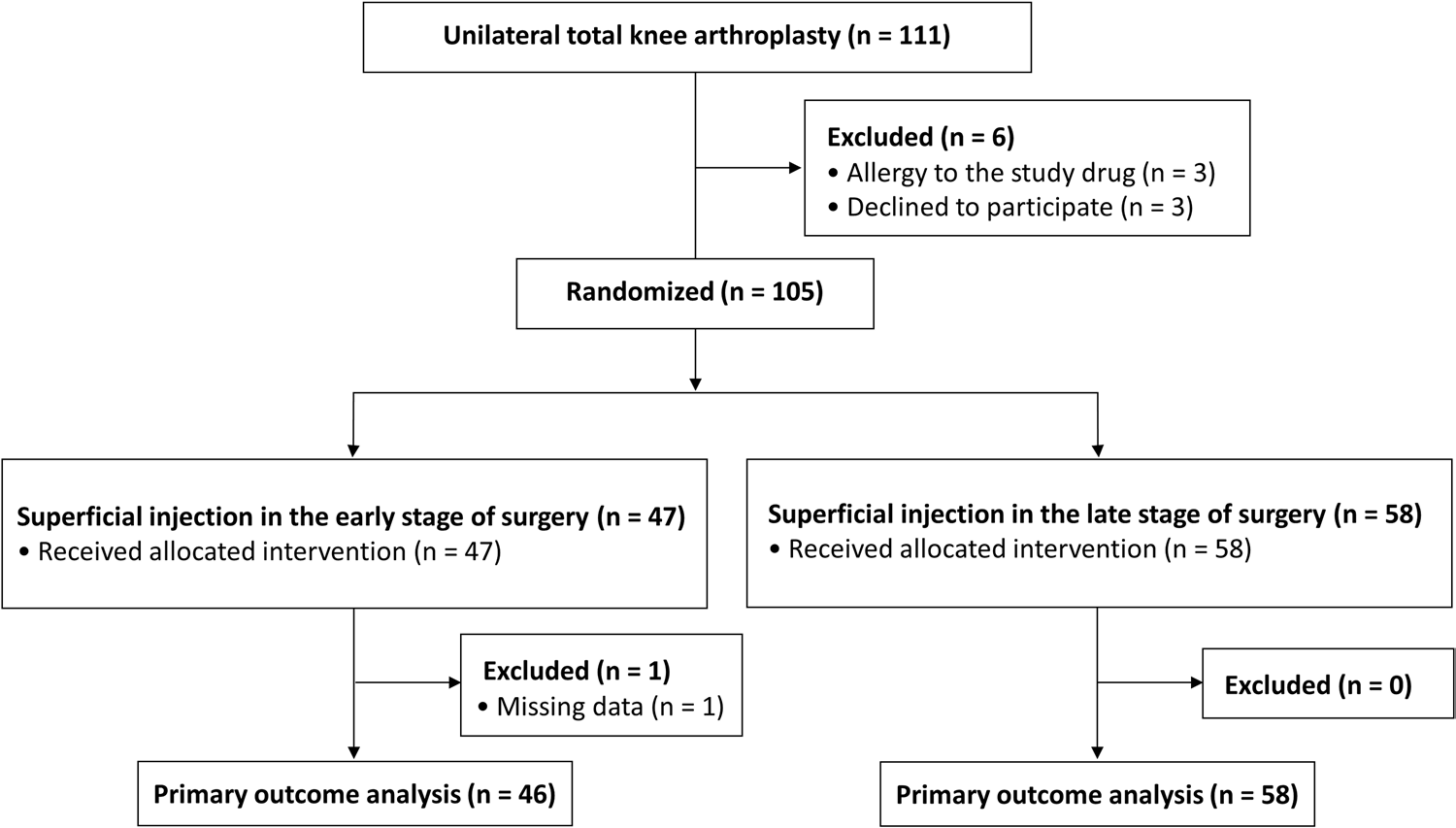
Detail of the enrollment and randomization of patients undergoing total knee arthroplasty. In both groups, deep injection was performed according to the same schedule

Figure 1 shows the flow chart of participants through the study. All randomized patients received superficial injection according to the allocated timing. One patient in the early stage periarticular injection group was excluded from the analysis of primary outcome because of missing data regarding pain VAS score at the recovery room due to postoperative delirium.

Table 1 summarizes the demographic characteristics of patients. Preoperative knee flexion angle was significantly inferior in the early stage periarticular injection group than the late-stage periarticular injection group (113° ± 21° versus 121° ± 15°; *p* = 0.025).

**Table 1:** Patient demographic and baseline clinical characteristics

	Superficial injection in the early stage of surgery (*n* = 47)	Superficial injection in the late stage of surgery (*n* = 58)	*p* value
Age, years	76 ± 8	74 ± 9	n.s.*
Sex (female/male)	36/11	45/13	n.s.^†^
Side (right/left)	21/26	24/34	n.s.^†^
Height (cm)	153 ± 10	153 ± 8	n.s.*
Weight (kg)	60.2 ± 12.1	60.4 ± 9.2	n.s.*
Body mass index, kg/m^2^	25.7 ± 3.5	26.1 ± 4.2	n.s.*
Preoperative diagnosis (OA/RA/AVN)	44/2/1	52/3/3	n.s.^†^
History of diabetes mellitus (yes/no)	9/38	6/52	n.s.^†^
Preoperative VAS at rest (mm)	30 ± 27	32 ± 28	n.s.*
Preoperative flexion angle (°)	113 ± 21	121 ± 15	0.025*
Preoperative extension angle (°)	− 12 ± 6	− 10 ± 7	n.s.*
Surgical approach (subvastus/lateral parapatellar)	47/0	57/1	n.s.^†^
Duration of operation (min)	99 ± 15	99 ± 15	n.s.*
Interval between superficial injection and transfer to recovery room (min)	105 ± 13	36 ± 5	< 0.0001*
Interval between deep injection and transfer to recovery room (min)	49 ± 4	50 ± 5	n.s.*

Results are shown as means ± standard deviation

*AVN* avascular necrosis; *OA* osteoarthritis; *RA* rheumatoid arthritis; *VAS* visual analog scale

*p values were determined with Student’s *t* test

^†^*p* values were determined with the Chi-squared test

### Primary outcome

The pain VAS score at recovery room was significantly lower in the early stage periarticular injection group than the late-stage periarticular injection group (23 ± 25 mm versus 39 ± 34 mm, respectively; mean difference 16 mm; 95% confidence interval 4–28 mm; *p* = 0.0078) (Table 2). The significance of difference was also confirmed with non-parametric statistics (median 13 mm versus 31 mm, *p* = 0.017, Mann–Whitney *U* test). The mean difference in primary outcome exceeded the level of clinical significance as indicated by the threshold value of MCID of 10 mm. The mean value of the primary outcome was below the threshold for PASS of 33 mm in the early stage periarticular injection group, but not in the late-stage periarticular injection group.

**Table 2:** Visual analog scale scores for postoperative pain at rest

	Superficial injection in the early stage of surgery (*n* = 47)	Superficial injection in the late stage of surgery (*n* = 58)	Difference (95% confidence interval)	*p* value
Recovery room	23 ± 25	39 ± 34	16 (4 to 28)	0.0078*
3 h after surgery	19 ± 19	24 ± 23	5 (− 3 to 13)	n.s.*
6 h after surgery	14 ± 16	15 ± 16	1 (− 5 to 8)	n.s.*
9 h after surgery	16 ± 23	17 ± 21	1 (− 8 to 9)	n.s.*
12 h after surgery	14 ± 19	15 ± 16	1 (− 7 to 7)	n.s.*
24 h after surgery	32 ± 25	35 ± 26	3 (− 7 to 13)	n.s.*
48 h after surgery	34 ± 26	30 ± 25	− 4 (− 15 to 6)	n.s.*

Visual analog scale score was rated using a 100-mm horizontal scale

Results are shown as means ± standard deviation

*p values were determined with Student’s *t* test

### Secondary outcomes

There were no significant differences between groups in terms of the pain VAS scores at 3 h, 6 h, 9 h, 12 h, 24 h, and 48 h after surgery (Table 2).

Intraoperative blood loss was significantly lower in the early stage periarticular injection group than the late-stage periarticular injection group (123 ± 47 mL versus 174 ± 102 mL, respectively; mean difference 51 mL; 95% confidence interval, 19–83 mL; *p* = 0.0021).

There were no significant differences in terms of the intraoperative consumption of remifentanil and fentanyl citrate between the early stage periarticular injection group and the late-stage periarticular injection group (Table 3).

**Table 3:** Intraoperative intravenous opioid consumption

	Superficial injection in the early stage of surgery (*n* = 47)	Superficial injection in the late stage of surgery (*n* = 58)	Difference (95% confidence interval)	*p* value
Remifentanil (mg)	2.0 ± 0.6	2.1 ± 0.6	0.1 (− 0.1 to 0.3)	n.s.*
Fentanyl citrate (µg)	81 ± 52	79 ± 55	− 2 (− 24 to 18)	n.s.*

Results are shown as means ± standard deviation

*p values were determined with Student’s *t* test

Table 4 summarizes the mean amounts of rescue analgesia. There was no significant difference in rescue analgesia between the two groups.

**Table 4:** Number of suppositories used as rescue analgesia

	Superficial injection in the early stage of surgery (*n* = 47)	Superficial injection in the late stage of surgery (*n* = 58)	Difference (95% confidence interval)	*p* value
On the night of surgery	0.3 ± 0.6	0.3 ± 0.5	0 (− 0.2 to 0.2)	n.s.*
Postoperative day 1	0.6 ± 0.8	0.5 ± 0.6	− 0.1 (− 0.4 to 0.1)	n.s.*
Postoperative day 2	0.6 ± 0.8	0.5 ± 0.6	− 0.1 (− 0.4 to 0.1)	n.s.*
Postoperative day 3	0.3 ± 0.6	0.4 ± 0.5	0.1 (− 0.3 to 0.1)	n.s.*

Results are shown as means ± standard deviation

*p values were determined with Student’s *t* test

The range of knee movement is shown in Table 5. No significant differences were observed between the two groups from days 1 to 7 after TKA.

**Table 5:** Range of movement following total knee arthroplasty

	Superficial injection in the early stage of surgery (*n* = 47)	Superficial injection in the late stage of surgery (*n* = 58)	Difference (95% confidence interval)	*p* value
Flexion angle				
Postoperative day 1	92 ± 13	94 ± 12	2 (− 2 to 7)	n.s.*
Postoperative day 2	97 ± 13	100 ± 11	3 (− 2 to 7)	n.s.*
Postoperative day 3	103 ± 10	103 ± 11	0 (− 4 to 5)	n.s.*
Postoperative day 4	106 ± 10	107 ± 12	1 (− 3 to 5)	n.s.*
Postoperative day 5	108 ± 10	109 ± 11	1 (− 3 to 5)	n.s.*
Postoperative day 6	109 ± 11	110 ± 10	1 (− 3 to 5)	n.s.*
Postoperative day 7	110 ± 10	112 ± 10	2 (− 3 to 5.3)	n.s.*
Extension angle				
Postoperative day 1	− 6 ± 6	− 6 ± 4	0 (− 2 to 2)	n.s.*
Postoperative day 2	− 5 ± 4	− 5 ± 4	0 (− 1 to 2)	n.s.*
Postoperative day 3	− 3 ± 3	− 4 ± 3	− 1 (− 1 to 1)	n.s.*
Postoperative day 4	− 3 ± 2	− 3 ± 3	0 (0 to 2)	n.s.*
Postoperative day 5	− 2 ± 2	− 3 ± 3	− 1 (− 2 to 0)	n.s.*
Postoperative day 6	− 2 ± 3	− 2 ± 2	0 (− 1 to 1)	n.s.*
Postoperative day 7	− 1 ± 2	− 2 ± 3	− 1 (− 1 to 1)	n.s.*

Results are shown as means ± standard deviation

*p values were determined with Student’s *t* test

Table 6 summarizes complications. There were no significant differences between the two groups.

**Table 6:** Complications

	Superficial injection in the early stage of surgery (*n* = 47)	Superficial injection in the late stage of surgery (*n* = 58)	*p* value
Postoperative nausea (yes/no)	14/33	22/36	n.s.^†^
Transient peroneal nerve palsy (yes/no)	0/47	1/57	n.s.^†^
Deep surgical site infection (yes/no)	0/47	0/58	n.s.^†^
Delayed wound healing (yes/no)	1/46	2/56	n.s.^†^
Proximal deep venous thrombosis (yes/no)	0/47	0/58	n.s.^†^
Distal deep venous thrombosis (yes/no)	4/43	1/57	n.s.^†^

^†^*p* values were determined with the Chi-squared test

## Discussion

The most important finding of this study was that periarticular injection performed in the early stage of surgery reduced postoperative pain at the recovery room compared with periarticular injection performed in the late stage of surgery during TKA managed under general anesthesia without regional block. The effect size fulfilled the MCID.

The statistically significant and clinically important difference in the improvement of postoperative pain was observed only at recovery room. This short-term difference would be beneficial for patients, because this improvement required only the timing of periarticular injection to be brought forward. The previous studies indicated that periarticular injection including ropivacaine, morphine, methylprednisolone, ketoprofen, and epinephrine provided more long-term pain relief [21, 22]. The pharmacokinetics that the agents included in the periarticular injection are not rapid-acting may affect this short-term difference between the two groups, because the mean interval between periarticular injection and transfer to the recovery room was short (50 min) in the late-stage periarticular injection group [23, 24]. The periarticular injection may require more time to provide sufficient analgesia.

The mean amount of intraoperative blood loss in the early periarticular injection group was approximately 50 mL less than that in the late periarticular injection group. Although statistically significant, this difference may be no clinically important. This difference may have been, because the periarticular injection solution included epinephrine and surgery was performed without use of a pneumatic tourniquet.

The levels of intraoperative remifentanil and fentanyl citrate consumption did not differ between the early stage periarticular injection group and the late-stage periarticular injection group. This was speculated to have been, because the intraoperative noxious stimuli were mainly derived from bone and periosteum. As the periarticular injection could reduce the postoperative pain derived from soft tissues, there would be no difference in terms of the amount of intraoperative opioid consumption.

The combined general anesthesia and periarticular injection regimen is an attractive option as it avoids the risk of nerve injury, although several investigators suggested that there has been a shift away from general anesthesia to regional anesthesia [10, 12]. Especially, the combined general anesthesia and periarticular injection regimen is beneficial for patients receiving anticoagulant therapy. Earlier periarticular injection than the conventional timing would be recommended for patients undergoing TKA managed with general anesthesia.

The most important limitation of our study was the lack of blinding for the surgical team and anesthesiologist. Although sham injection using normal saline could be performed, sham injection was abandoned to minimize damage to soft tissues around knee joint. Wound-healing problems after TKA were shown to be associated with increased risk of further complications [4].

Although the threshold value of MCID used in the present study was based on a previous detailed study [14], we should note that there have been no studies to determine the threshold value of MCID in VAS pain score in Asian populations following TKA. Similarly, although the test–retest reliability of VAS pain score has been reported to be substantial or almost perfect in patients with acute pain [1, 14], there have been no reports regarding its reliability in Asian populations undergoing TKA.

With regard to clinical relevance, this study suggested that the early stage periarticular injection may be associated with preferable postoperative pain relief than late-stage periarticular injection in patients undergoing TKA managed with general anesthesia.

## Conclusions

Superficial injection just prior to arthrotomy provided clinically meaningful improvement in pain following TKA managed under general anesthesia without regional block and a modest decrease in intraoperative blood loss compared with superficial injection after completing total knee prosthesis implantation.

## References

[1] Bijur PE, Silver W, Gallagher EJ (2001). Reliability of the visual analog scale for measurement of acute pain. Acad Emerg Med.

[2] Busch CA, Shore BJ, Bhandari R, Ganapathy S, MacDonald SJ, Bourne RB, Rorabeck CH, McCalden RW (2006). Efficacy of periarticular multimodal drug injection in total knee arthroplasty. A randomized trial. J Bone Jt Surg Am.

[3] Crile GW (1913). The kinetic theory of shock and its prevention through anoci-association (shockless operation). Lancet.

[4] Galat DD, McGovern SC, Larson DR, Harrington JR, Hanssen AD, Clarke HD (2009). Surgical treatment of early wound complications following primary total knee arthroplasty. J Bone Jt Surg Am.

[5] Hirasawa N, Kurosaka K, Nishino M, Nakayama T, Matsubara M, Tsukada S (2018). No clinically important difference in pain scores after THA between periarticular analgesic injection and placebo: a randomized trial. Clin Orthop Relat Res.

[6] Jaeschke R, Singer J, Guyatt GH (1989). Measurement of health status. Ascertaining the minimal clinically important difference. Control Clin Trials.

[7] Kanda Y (2013). Investigation of the freely available easy-to-use software ‘EZR’ for medical statistics. Bone Marrow Transpl.

[8] Kissin I (2000). Preemptive analgesia. Anesthesiology.

[9] Kurosaka K, Tsukada S, Nakayama H, Iseki T, Kanto R, Sugama R, Yoshiya S (2018). Periarticular injection versus femoral nerve block for pain relief after anterior cruciate ligament reconstruction: a randomized controlled trial. Arthroscopy.

[10] Lee GC (2017). What’s new in adult reconstructive knee surgery. J Bone Jt Surg Am.

[11] Little RJ, D’Agostino R, Cohen ML, Dickersin K, Emerson SS, Farrar JT, Frangakis C, Hogan JW, Molenberghs G, Murphy SA, Neaton JD, Rotnitzky A, Scharfstein D, Shih WJ, Siegel JP, Stern H (2012). The prevention and treatment of missing data in clinical trials. N Engl J Med.

[12] Maheshwari AV, Blum YC, Shekhar L, Ranawat AS, Ranawat CS (2009). Multimodal pain management after total hip and knee arthroplasty at the Ranawat Orthopaedic Center. Clin Orthop Relat Res.

[13] Murata-Ooiwa M, Tsukada S, Wakui M (2017). Intravenous acetaminophen in multimodal pain management for patients undergoing total knee arthroplasty: a randomized, double-blind, placebo-controlled trial. J Arthroplasty.

[14] Myles PS, Myles DB, Galagher W, Boyd D, Chew C, MacDonald N, Dennis A (2017). Measuring acute postoperative pain using the visual analog scale: the minimal clinically important difference and patient acceptable symptom state. Br J Anaesth.

[15] Ong CK, Lirk P, Seymour RA, Jenkins BJ (2005). The efficacy of preemptive analgesia for acute postoperative pain management: a meta-analysis. Anesth Analg.

[16] Ranawat AS, Ranawat CS (2007). Pain management and accelerated rehabilitation for total hip and total knee arthroplasty. J Arthroplasty.

[17] Saito M, Tsukada S, Fujita N, Rahman M, Morita W, Kitamura N, Tasaki A (2018). Post-operative pain control following arthroscopic rotator cuff repair: peri-articular injection versus interscalene brachial plexus block. Int Orthop.

[18] Spangehl MJ, Clarke HD, Hentz JG, Misra L, Blocher JL, Seamans DP (2015). The Chitranjan Ranawat Award: periarticular injections and femoral & sciatic blocks provide similar pain relief after TKA: a randomized clinical trial. Clin Orthop Relat Res.

[19] Tashjian RZ, Deloach J, Porucznik CA, Powell AP (2009). Minimal clinically important differences (MCID) and patient acceptable symptomatic state (PASS) for visual analog scales (VAS) measuring pain in patients treated for rotator cuff disease. J Shoulder Elbow Surg.

[20] Tsukada S, Kurosaka K, Nishino M, Hirasawa N (2018). Cutaneous hypesthesia and kneeling ability after total knee arthroplasty: a randomized controlled trial comparing anterolateral and anteromedial skin incision. J Arthroplasty.

[21] Tsukada S, Wakui M, Hoshino A (2015). Pain control after simultaneous bilateral total knee arthroplasty: a randomized controlled trial comparing periarticular injection and epidural analgesia. J Bone Jt Surg Am.

[22] Tsukada S, Wakui M, Hoshino A (2014). Postoperative epidural analgesia compared with intraoperative periarticular injection for pain control following total knee arthroplasty under spinal anesthesia: a randomized controlled trial. J Bone Jt Surg Am.

[23] Tsukada S, Wakui M, Hoshino A (2016). The impact of including corticosteroid in a periarticular injection for pain control after total knee arthroplasty: a double-blind randomised controlled trial. Bone Jt J.

[24] Vendittoli PA, Makinen P, Drolet P, Lavigne M, Fallaha M, Guertin MC, Varin F (2006). A multimodal analgesia protocol for total knee arthroplasty. A randomized, controlled study. J Bone Jt Surg Am.

[25] Yadeau JT, Goytizolo EA, Padgett DE, Liu SS, Mayman DJ, Ranawat AS, Rade MC, Westrich GH (2013). Analgesia after total knee replacement: local infiltration versus epidural combined with a femoral nerve blockade: a prospective, randomised pragmatic trial. Bone Jt J.

